# Draft Genome Sequence of a Cellulase-Producing Psychrotrophic Paenibacillus Strain, IHB B 3415, Isolated from the Cold Environment of the Western Himalayas, India

**DOI:** 10.1128/genomeA.01581-14

**Published:** 2015-02-19

**Authors:** Hena Dhar, Mohit Kumar Swarnkar, Arvind Gulati, Anil Kumar Singh, Ramesh Chand Kasana

**Affiliations:** a Hill Area Tea Science Division, CSIR-Institute of Himalayan Bioresource Technology, Palampur, India; b Academy of Scientific and Innovative Research, New Delhi, India; c Division of Biotechnology, CSIR-Institute of Himalayan Bioresource Technology, Palampur, India

## Abstract

Paenibacillus sp. strain IHB B 3415 is a cellulase-producing psychrotrophic bacterium isolated from a soil sample from the cold deserts of Himachal Pradesh, India. Here, we report an 8.44-Mb assembly of its genome sequence with a G+C content of 50.77%. The data presented here will provide insights into the mechanisms of cellulose degradation at low temperature.

## GENOME ANNOUNCEMENT

Low**-**temperature environments are inhabited by cold-adapted microorganisms, psychrotrophs and psychrophiles. These cold-adapted microorganisms are sources of commercially and industrially important enzymes, including cellulases (1). During our exploration of the microbial diversity associated with the soils of cold environments of Himachal Pradesh, India, we isolated a Paenibacillus strain, IHB B 3415, which produces cellulase at low temperature. This bacterium appears to be most closely related to Paenibacillus borealis KK19^T^ based on a 16S rRNA gene sequence analysis. The genome of Paenibacillus sp. IHB B 3415 was sequenced, given the ability of the organism to grow and produce low**-**temperature active enzymes.

Whole-genome shotgun sequencing was completed using the Illumina Genome Analyzer IIx in 76-bp paired-read format. A partial flow cell obtained 28,321,562 raw paired-end (PE) reads with 4,304,877,424 bases of raw sequence. The paired reads were quality filtered using the NGS QC toolkit version 2.3 (2) (cutoff read length for HQ, 70%; cutoff quality score, 20). A sum of 23,383,467 (77.57%) of the filtered PE reads were obtained without adaptor/primer contamination and were used further for assembly. *De novo* assembly of the genome data was done using Velvet version 1.2.10 (3). In this data set, the *k* parameter (from 49 to 73) was optimized for best assembly. We found that at *k* of 51 mers, 89.5% (41,853,238) of the reads were aligned out of 46,766,934 total reads. The Paenibacillus sp. IHB B 3415 genome was assembled in 752 contigs, with a sum of 8,350,804 bases (*N*_50_ length, 26,240 bases; maximum contig length, 110 kb). We used SSPACE version 3.0 (4) to extend and merge the resulting scaffolds based on read-pair information and short overlaps to reduce the number of scaffolds. GapFiller version 1.1 (5) was used to close the gaps between short scaffolds contained within the large scaffolds by replacing unknown nucleotides (Ns) with true nucleotides based on paired-read information and short overlaps. After filling the gaps, the reads were assembled as 290 scaffolds summing 8,437,849 bp (*N*_50_ size, 78,530 bp; longest size, 315,231 bp; G+C content, 50.77%). Annotation conducted on the RAST server using the Glimmer3 option predicted 7,897 protein-coding genes, including 78 RNA genes and 454 predicted SEED subsystem features (6). A total of 2,868 (37%) features were covered by SEED subsystems, out of which 2,737 were nonhypothetical proteins. The annotation of Paenibacillus sp. IHB B 3415 using Prodigal (7) predicted 7,335 coding sequences summing to a total of 7,161,309 bases, which is ~85% of the assembled size of the genome (8,437,849 bases). Further, tRNAscan-SE (8) and RNAmmer (9) predicted 77 noncoding RNAs (71 tRNAs, 1 pseudo-tRNA, and 3 RNAs consisting of 3 copies of 5S rRNA and one copy each of 16S rRNA and 23S rRNA genes).

It is interesting to note that the genome of Paenibacillus sp. IHB B 3415 is currently the second largest genome in the genus after Paenibacillus mucilaginosus in both overall size and the number of genes encoding proteins (10). In the IHB B 3415 genome, 1,011 genes were assigned for carbohydrate metabolism, 453 for amino acids and derivatives, and 131 for stress response (including 18 and 3 for heat and cold shock, respectively). The genome contains 16 genes predicted for cellulases, corroborating our results for cellulose degradation effected by the strain. A comparative genome analysis of cellulase-producing psychrotrophic Paenibacillus sp. IHB B 3415 with other Paenibacillus species will give insight into the evolutionary relationships and biotechnological significance of this important genus.

### Nucleotide sequence accession numbers.

This whole-genome shotgun project has been deposited at DDBJ/EMBL/GenBank under the accession no. JUEI00000000. The version described in this paper is version JUEI01000000.

## References

[1] Kasana RC, Gulati A 2011 Cellulases from psychrophilic microorganisms: a review. J Basic Microbiol 51:572–579. doi:10.1002/jobm.201000385. 21656807

[2] Patel RK, Jain M 2012 NGS QC toolkit: a toolkit for quality control of next generation sequencing data. PLoS One 7:e30619. doi:10.1371/journal.pone.0030619. 22312429 PMC3270013

[3] Zerbino DR, Birney E 2008 Velvet: algorithms for *de novo* short read assembly using de Bruijn graphs. Genome Res 18:821–829. doi:10.1101/gr.074492.107. 18349386 PMC2336801

[4] Boetzer M, Henkel CV, Jansen HJ, Butler D, Pirovano W 2011 Scaffolding pre-assembled contigs using SSPACE. Bioinformatics 27:578–579. doi:10.1093/bioinformatics/btq683. 21149342

[5] Boetzer M, Pirovano W 2012 Toward almost closed genomes with GapFiller. Genome Biol 13:R56. doi:10.1186/gb-2012-13-6-r56. 22731987 PMC3446322

[6] Aziz RK, Bartels D, Best AA, DeJongh M, Disz T, Edwards RA, Formsma K, Gerdes S, Glass EM, Kubal M, Meyer F, Olsen GJ, Olson R, Osterman AL, Overbeek RA, McNeil LK, Paarmann D, Paczian T, Parrello B, Pusch GD, Reich C, Stevens R, Vassieva O, Vonstein V, Wilke A, Zagnitko O 2008 The RAST server: Rapid Annotations using Subsystems Technology. BMC Genomics 9:75. doi:10.1186/1471-2164-9-75. 18261238 PMC2265698

[7] Hyatt D, Chen GL, Locascio PF, Land ML, Larimer FW, Hauser LJ 2010 Prodigal: prokaryotic gene recognition and translation initiation site identification. BMC Bioinformatics 11:119. doi:10.1186/1471-2105-11-119. 20211023 PMC2848648

[8] Lowe TM, Eddy SR 1997 tRNAscan-SE: a program for improved detection of transfer RNA genes in genomic sequence. Nucleic Acids Res 25:955–964. doi:10.1093/nar/25.5.0955. 9023104 PMC146525

[9] Lagesen K, Hallin PF, Rødland EA, Staerfeldt HH, Rognes T, Ussery DW 2007 RNammer: consistent annotation of ribosomal RNA genes. Nucleic Acids Res 35:3100–3108. doi:10.1093/nar/gkm160. 17452365 PMC1888812

[10] Lu JJ, Wang JF, Hu XF 2013 Genome sequence of growth-improving *Paenibacillus mucilaginosus* strain knp414. Genome Announc 1(5):e00881-13. doi:10.1128/genomeA.00881-13. 24158556 PMC3813186

